# Supplementary material for: Regional variations in childbirth interventions and their correlations with adverse outcomes, birthplace and care provider: A nationwide explorative study
Source: PLoS One. 2020 Mar 5;15(3):e0229488. doi: 10.1371/journal.pone.0229488 (PMC7058301; doi:10.1371/journal.pone.0229488)
Supplement: S1 Table — *odds ratios, adjusted for parity, maternal age, ethnic background, socioeconomic position and urbanisation. (DOCX) [file pone.0229488.s001.docx]

**S3 Table. Multivariable logistic regression of process of care and intervention rates by region, adjusted* OR [99% CI]**

| ***All women*** | | | | | | | | | | | | |
| --- | --- | --- | --- | --- | --- | --- | --- | --- | --- | --- | --- | --- |
|  | **GR** | **FR** | **DR** | **OV** | **FL** | **GD** | **UT** | **NH** | **ZH** | **ZL** | **NB** | **LB** |
| **Total *n*** | 19,441 | 22,568 | 15,875 | 42,869 | 17,461 | 71,286 | 52,893 | 105,948 | 139,573 | 11,327 | 84,187 | 31,302 |
| **Women in midwife-led care at onset of labour** | 0.94  [0.90-0.97] | 1.16  [1.12-1.20] | 0.84  [0.80-0.87] | 1.12  [1.09-1.15] | 0.91  [0.87-0.94] | 1.23  [1.20-1.25] | 1.26  [1.23-1.29] | 1.21  [1.19-1.23] | 0.91  [0.89-0.93] | 0.99  [0.94-1.04] | 0.87  [0.85-0.89] | 0.75  [0.73-0.77] |
| **Women in midwife-led care at time of birth** | 0.98  [0.94-1.01] | 1.15  [1.11-1.19] | 0.81  [0.77-0.85] | 1.20  [1.17-1.23] | 0.83  [0.79-0.87] | 1.19  [1.16-1.21] | 1.12  [1.10-1.15] | 1.16  [1.14-1.19] | 1.05  [1.03-1.07] | 1.12  [1.06-1.17] | 0.77  [0.76-0.79] | 0.79  [0.77-0.82] |
| **Planned home birth** | 1.05  [1.001-1.10] | 1.74  [1.67-1.81] | 0.98  [0.93-1.03] | 1.59  [1.54-1.63] | 1.10  [1.04-1.16] | 1.51  [1.48-1.55] | 1.13  [1.10-1.17] | 1.18  [1.15-1.21] | 0.96  [0.93-0.98] | 0.36  [0.33-0.39] | 0.84  [0.82-0.86] | 0.56  [0.53-0.58] |
| **Actual home birth** | 1.08  [1.03-1.14] | 1.45  [1.39-1.51] | 0.85  [0.80-0.90] | 1.40  [1.36-1.45] | 1.08  [1.02-1.14] | 1.44  [1.41-1.48] | 1.08  [1.04-1.12] | 1.20  [1.17-1.23] | 0.93  [0.90-0.95] | 0.55  [0.52-0.60] | 0.75  [0.72-0.77] | 0.70  [0.67-0.73] |
| **Episiotomy in vaginal births** | 0.79  [0.75-0.83] | 0.87  [0.83-0.91] | 1.03  [0.98-1.08] | 1.14  [1.10-1.18] | 0.70  [0.67-0.74] | 0.98  [0.95-1.002] | 0.81  [0.78-0.83] | 0.71  [0.69-0.73] | 1.10  [1.07-1.12] | 1.54  [1.45-1.63] | 1.35  [1.31-1.38] | 1.40  [1.35-1.45] |
| ***Women in midwife-led care at onset of labour*** | | | | | | | | | | | | |
|  | **GR** | **FR** | **DR** | **OV** | **FL** | **GD** | **UT** | **NH** | **ZH** | **ZL** | **NB** | **LB** |
| **Total *n*** | 10,013 | 12,901 | 7,884 | 23,922 | 8,942 | 41,401 | 30,601 | 59,750 | 70,023 | 6,068 | 42,040 | 14,464 |
| **Intrapartum referral to obstetrician-led care** | 0.96  [0.91-1.01] | 0.93  [0.88-0.97] | 1.17  [1.10-1.24] | 0.84  [0.81-0.88] | 1.21  [1.14-1.29] | 0.96  [0.93-0.99] | 1.07  [1.04-1.11] | 0.97  [0.94-0.99] | 0.83  [0.81-0.85] | 0.80  [0.75-0.86] | 1.32  [1.29-1.36] | 1.09  [1.04-1.14] |
| **Artificial rupture of membranes** | 0.90  [0.86-0.95] | 1.00  [0.96-1.05] | 1.05  [0.99-1.12] | 1.03  [0.99-1.12] | 1.05  [0.99-1.12] | 0.97  [0.95-1.00] | 0.95  [0.92-0.98] | 1.00  [0.98-1.03] | 1.08  [1.05-1.11] | 1.10  [1.03-1.17] | 1.02  [0.99-1.05] | 0.87  [0.84-0.91] |
| ***Women in midwife-led care at time of birth*** | | | | | | | | | | | | |
|  | **GR** | **FR** | **DR** | **OV** | **FL** | **GD** | **UT** | **NH** | **ZH** | **ZL** | **NB** | **LB** |
| **Total *n*** | 5,912 | 7,985 | 4,461 | 14,962 | 4,706 | 24,600 | 17,014 | 33,578 | 42,223 | 3,922 | 21,557 | 8,140 |
| **Oxytocin postpartum** | 0.42  [0.40-0.45] | 0.72  [0.68-0.77] | 0.95  [0.88-1.03] | 0.66  [0.63-0.69] | 1.17  [1.07-1.28] | 0.69  [0.67-0.72] | 1.16  [1.11-1.22] | 0.80  [0.78-0.83] | 1.15  [1.10-1.19] | 2.37  [2.12-2.66] | 1.44  [1.38-1.51] | 1.77  [1.64-1.90] |
| **Episiotomy** | 0.69  [0.61-0.77] | 0.78  [0.70-0.86] | 1.08  [0.96-1.21] | 1.43  [1.34-1.52] | 0.50  [0.42-0.59] | 0.99  [0.93-1.05] | 0.81  [0.75-0.87] | 0.62  [0.58-0.65] | 1.22  [1.16-1.28] | 2.04  [1.84-2.26] | 1.40  [1.33-1.48] | 1.42  [1.31-1.54] |
| ***Women in obstetrician-led care at time of birth*** | | | | | | | | | | | | |
|  | **GR** | **FR** | **DR** | **OV** | **FL** | **GD** | **UT** | **NH** | **ZH** | **ZL** | **NB** | **LB** |
| **Total *n*** | 13,477 | 14,512 | 11,375 | 27,678 | 12,712 | 46,372 | 35,665 | 71,249 | 96,023 | 7,368 | 62,386 | 23,030 |
| **Episiotomy in vaginal births** | 0.80  [0.76-0.85] | 0.95  [0.90-1.01] | 0.95  [0.90-1.01] | 1.14  [1.10-1.19] | 0.69  [0.65-0.73] | 1.03  [0.99-1.06] | 0.82  [0.79-0.85] | 0.76  [0.74-0.78] | 1.09  [1.06-1.12] | 1.49  [1.39-1.60] | 1.26  [1.23-1.30] | 1.34  [1.28-1.39] |

*odds ratios, adjusted for parity, maternal age, ethnic background, socioeconomic position and urbanisation.
