# Supplementary material for: Regional variations in childbirth interventions and their correlations with adverse outcomes, birthplace and care provider: A nationwide explorative study
Source: PLoS One. 2020 Mar 5;15(3):e0229488. doi: 10.1371/journal.pone.0229488 (PMC7058301; doi:10.1371/journal.pone.0229488)
Supplement: S2 Table — All correlations are based on adjusted ORs of the intervention rates (adjusted for parity, maternal age, ethnic background, socioeconomic position and urbanisation). A p-value of 0.05 corresponds with a correlation of rho ≥ 0.57 or ≤ - 0.57 (95% confidence intervals 0.001–0.86). Since the sample size for all measured correlations is the same, namely 12 regions, the correlation is significant at the same value of rho for all measured correlations. Correlations with rho ≥ 0.60 or ≤ - 0.60 are indicated in bold type since they are considered strong. (DOCX) [file pone.0229488.s002.docx]

**S4 Table. Correlations between process of care variables and interventions**

|  | ***Women in midwife-led care at onset of labour*** | ***Intrapartum referral to obstetrician-led care*** | ***Planned home birth*** | ***Actual home birth*** | ***Episiotomy in midwife-led care at time of birth*** | ***Episiotomy in all births*** | ***Oxytocin postpartum*** |
| --- | --- | --- | --- | --- | --- | --- | --- |
| ***Women in midwife-led care at time of birth*** | **rho = 0.84** | **rho = - 0.66** | **rho = 0.73** | **rho = 0.73** | rho = - 0.06 | rho = - 0.20 | rho = - 0.57 |
| ***Intrapartum referral to obstetrician-led care*** | rho = - 0.40 |  | rho = - 0.15 | rho = - 0.21 | rho = - 0.33 | rho = - 0.26 | rho = 0.28 |
| ***Planned home*** | **rho = 0.69** |  |  | **rho = 0.98** | rho = - 0.48 | rho = - 0.57 | **rho = - 0.75** |
| ***Actual home birth*** | **rho = 0.69** |  |  |  | rho = - 0.53 | **rho = - 0.60** | **rho = - 0.79** |
| ***Episiotomy in midwife-led care at time of birth*** | rho = - 0.31 |  |  |  |  |  | rho = 0.37 |
| ***Episiotomy in obstetrician-led care at time of birth*** |  | rho = - 0.31 |  |  | **rho = 0.96** |  |  |
| ***Artificial rupture of membranes*** | rho = - 0.21 | rho = - 0.27 | rho = - 0.24 | rho = - 0.27 | rho = 0.28 |  | rho = 0.29 |

All correlations are based on adjusted ORs of the intervention rates (adjusted for parity, maternal age, ethnic background, socioeconomic position and urbanisation).

A p-value of 0.05 corresponds with a correlation of rho ≥ 0.57 or ≤ - 0.57 (95% confidence intervals 0.001-0.86).

Since the sample size for all measured correlations is the same, namely 12 regions, the correlation is significant at the same value of rho for all measured correlations. Correlations with rho ≥ 0.60 or ≤ - 0.60 are indicated in bold type since they are considered strong.
