# Supplementary material for: Regional variations in childbirth interventions and their correlations with adverse outcomes, birthplace and care provider: A nationwide explorative study
Source: PLoS One. 2020 Mar 5;15(3):e0229488. doi: 10.1371/journal.pone.0229488 (PMC7058301; doi:10.1371/journal.pone.0229488)
Supplement: S3 Table — All correlations are based on adjusted ORs of the intervention rates (adjusted for parity, maternal age, ethnic background, socioeconomic position and urbanisation). Correlations for still birth and mortality are not calculated since these were not significantly different between the regions. A p-value of 0.05 corresponds with a correlation of rho ≥ 0.57 or ≤ - 0.57 (95% confidence intervals 0.001–0.86). Since the sample size for all measured correlations is the same, namely 12 regions, the correlation is significant at the same value of rho for all measured correlations. Correlations with rho ≥ 0.60 or ≤ - 0.60 are indicated in bold type since they are considered strong. (DOCX) [file pone.0229488.s003.docx]

**S5 Table. Correlations between process of care variables or interventions, with obstetric outcomes**

| **Process of care and interventions in subgroups of:** | | **Obstetric outcomes for women in the same subgroup as the process of care or intervention subgroups$** | | |
| --- | --- | --- | --- | --- |
|  |  | *Apgar score below 7 at 5 minutes* | *3^rd^ and 4^th^ degree perineal tear for vaginal births* | *Postpartum haemorrhages ≥1000 ml* |
| **all women** | *Women in midwife-led care at onset of labour* | rho = 0.07 | rho = 0.11 | rho = - 0.37 |
|  | *Women in midwife-led care at time of birth* | rho = 0.01 | rho = 0.14 | rho = - 0.46 |
|  | *Planned home birth* | rho = 0.30 | rho = 0.45 | rho = - 0.15 |
|  | *Actual home birth* | rho = 0.46 | rho = 0.32 | rho = - 0.12 |
|  | *Episiotomy in vaginal births among all women* | rho = - 0.52 | rho = - 0.20 | rho = - 0.12 |
| **women in midwife-led care at onset of labour** | *Intrapartum referral to obstetrician-led care* | rho = - 0.05 | rho = 0.32 | **rho = 0.74** |
|  | *Artificial rupture of membranes* | rho = - 0.31 | rho = - 0.28 | rho = - 0.10 |
| **women in midwife-led care at time of birth** | *Oxytocin postpartum* | rho = - 0.50 | rho = - 0.36 | rho = 0.08 |

All correlations are based on adjusted ORs of the intervention rates (adjusted for parity, maternal age, ethnic background, socioeconomic position and urbanisation).

Correlations for still birth and mortality are not calculated since these were not significantly different between the regions.

A p-value of 0.05 corresponds with a correlation of rho ≥ 0.57 or ≤ - 0.57 (95% confidence intervals 0.001-0.86).

Since the sample size for all measured correlations is the same, namely 12 regions, the correlation is significant at the same value of rho for all measured correlations. Correlations with rho ≥ 0.60 or ≤ - 0.60 are indicated in bold type since they are considered strong.
