# Supplementary material for: Regional variations in childbirth interventions and their correlations with adverse outcomes, birthplace and care provider: A nationwide explorative study
Source: PLoS One. 2020 Mar 5;15(3):e0229488. doi: 10.1371/journal.pone.0229488 (PMC7058301; doi:10.1371/journal.pone.0229488)
Supplement: Attachment — Submitted filename: Response to Reviewers 19-18857.docx [file pone.0229488.s004.docx]

We previously published a related manuscript entitled ‘Regional variations in childbirth interventions

in the Netherlands: a nationwide explorative study’ in BMC Pregnancy and Childbirth (doi:

10.1186/s12884-018-1795-0).

**Academic editor comments:**

*Substantial overlap has been identified in your submitted manuscript and that or your previously*

*published work from this study. However, if you are able to substantiate that the outcomes and*

*exposures are substantially different between the two manuscripts a new submission may be*

*considered for publication in PLOS ONE. It appears that the primary outcomes in this manuscript are:*

*the number of women receiving midwife-led care at the onset of their pregnancy, at the onset of*

*labour, and at the time of birth; intrapartum referral to obstetrician-led care, and the planned, and*

*actual place of birth (home, hospital and birth centre midwife-led, hospital obstetrician-led). And the*

*primary outcomes in the previous publication are: induction of labour; augmentation after a*

*spontaneous onset of labour; intrapartum oxytocin use; epidural analgesia; other pharmacological*

*pain relief; instrumental vaginal birth; CS (prelabour, intrapartum); and involvement of a*

*paediatrician in the first 24 h after birth.*

*That being said, a revision of the existing manuscript is required. You will need to re-submit a new*

*submission for PLOS ONE. You need to give careful consideration and clear expose of how to*

*incorporate your previous findings into this manuscript with enhanced and clearly explicit*

*acknowledgement of the previous work and how this manuscript differs from it. For example, Table 1*

*is an exact duplicate of the previously published work and that is unacceptable. Either reference that*

*table in your submitted publication or present a table with more refined comparisons. The remainder*

*of your manuscript should be carefully checked for similar duplications and dealt with in the same*

*manner*.

We have removed table 1 with baseline characteristics and the S4 table with adverse outcomes from

our article and referred to our previous article in several lines in the manuscript.

Introduction, page 4, lines 92-94:

“Box 1 cites the description of the maternity care system in the Netherlands from our previous

publication on regional variations in the Netherlands (1).”

Introduction, page 5, lines 101-103:

“A previous article described regional variations in rates of induction and augmentation of labour,

pain medication, caesarean section, and involvement of paediatrician in the first 24 h after birth (1).”

Results, page 8, line 173:

“Maternal characteristics have previously been published (1).”

Results, page 11, lines 231-232:

Cover Letter

“Adverse neonatal and maternal outcomes have been described in a previous article (1).”

**Reviewer 4**

*For Data analysis, line 154-155, please provide details about the calculations about the weighted*

*overall rate of investigated outcomes. Is “region” included as independent categorical variable in the*

*logistic regression model or each region analyzed separately?*

Indeed, region was included as independent categorical variable in the logistic regression model. We

used the option ‘weighted mean’ as reference category in the analyses. We changed the sentence to

point out this method in our analyses.

Methods, page 7, lines 156-157:

“In the logistic regression analyses, region was included as independent variable and the overall rate

of the outcome we investigated, weighted for the number of women per region, was considered as a

reference.”

*Figure 3 legend, “number of” in “Adjusted OR of number of women…” should be removed.*

We removed these words.

*E.g line 310 “Lower rates were found in regions with more home births”. Such correlations seem*

*obvious because some interventions may not be made at home births. Will such correlation affect the*

*birth outcomes?*

In the Dutch maternity care system, episiotomies and postpartum administration of oxytocin can

both be used in home and hospital births. Therefore, it was a remarkable result in our study. We

added a sentence to point out the significance of this result.

Discussion, page 21, lines 309-310:

“These intervention can be applied in both home and hospital births, but lower rates were found in

regions with more home births.”

*Line 315-316, “The only correlation that we found with adverse neonatal and maternal outcomes was*

*between intrapartum referral and PPH; there were higher rates of PPH in regions with more*

*intrapartum referrals”. It is not surprised to see such correlation because intrapartum referral means*

*risk of adverse outcomes or complications arise. It worth looking at the reasons why some regions*

*had more intrapartum referrals.*

Previous studies of Offerhaus et al. (2015; doi: 0.1186/s12884-015-0471-x) and Schuit et al. (2016;

doi: 10.3109/14767058.2015.1124080) showed non-urgent referrals, such as meconium-stained

amniotic fluid, need for pain medication and/or delay in progress of first stage of labour, being the

main reasons for intrapartum referrals in the Netherlands. We believe it is an important finding that

intrapartum referral rates and PPH were correlated, because the main reasons for intrapartum

referrals do no justify a higher PPH risk. We added a note that intrapartum referral was defined as

referral after onset of labour but before birth.

Discussion, page 21, lines 315-317:

“The only correlation that we found with adverse neonatal and maternal outcomes was between

intrapartum referral and PPH; there were higher rates of PPH in regions with more intrapartum

referrals, which did not include referrals after birth.”

Discussion, page 23, lines 358-362:

“Offerhaus et al. (2015) described a similar correlation (15). Previous studies showed non-urgent

referrals, such as meconium-stained amniotic fluid, need for pain medication and/or delay in

progress of first stage of labour, being the main reasons for intrapartum referrals in the Netherlands

(15,25). Our findings of varying rates of intrapartum referral, which may be correlated with higher

PPH rates requires further investigation, before any conclusions in terms of policy with regard to

augmentation of labour may be drawn.”

*It would be more informative to examine what factors could have impact on the rates of the*

*investigated outcomes using multilevel modeling.*

We had the following considerations for not performing multilevel analyses.

The aim of our study is to describe and explore which variations exist and how variations in

interventions or processes relate to each other and to adverse outcomes. These variations might

(partly) be explained by clustering of women in care facilities (birth center, hospital, midwifery

practice) and care providers. Variations in clinical practice that are not explained by differences in

obstetric risks or preferences of women, may be unwarranted. If we would conduct multilevel

analyses, we would adjust for the differences between care providers or other levels. However, these

variations are exactly the topic of our study and we do not want to rule them out. Future research

should use multilevel analyses to investigate whether differences between care facilities and care

providers may explain the variations between regions, but that falls beyond the scope of our study.

Moreover, the size of our study population by region makes our assumption plausible that variations

in case mix (in terms of obstetric risks and preferences of women) are levelled out.

We are confident that our study is a first, important step in reducing the inappropriate use of medical

interventions in maternal and newborn care which is called for by important bodies, such as the

WHO. We therefore hope that you will consider publication of our article.

We look forward to hearing from you.

Yours faithfully, on behalf of all authors,

Anna Seijmonsbergen-Schermers, MSc
