# Supplementary material for: Regional variations in childbirth interventions and their correlations with adverse outcomes, birthplace and care provider: A nationwide explorative study
Source: PLoS One. 2020 Mar 5;15(3):e0229488. doi: 10.1371/journal.pone.0229488 (PMC7058301; doi:10.1371/journal.pone.0229488)
Supplement: Attachment — Submitted filename: summary of the report- Childbirth Interventions.docx [file pone.0229488.s005.docx]

# REGIONAL VARIATIONS IN CHILDBIRTH INTERVENTIONS AND THEIR CORRELATIONS WITH ADVERSE

# OUTCOMES, BIRTHPLACE AND CARE PROVIDER: A NATIONWIDE EXPLORATIVE STUDY

# SUMMARISED COMMENTS

| **Section** | **Comment, question, suggestion.** |
| --- | --- |
| **Abstract** | 1. There was no introduction in the background, the knowledge gap is missing, which could have been reflected in the introduction (i.e. this could have helped to understand why this study was important to be undertaken) 2. No study design is reported on methods 3. Methods not clearly stated 4. Missed the policy implications and recommendations in conclusion. |
| **Introduction**  **Background** | 1. Quantify the rates of interventions during childbirth and their variations globally to locally 2. What interventions have been done in Netherlands with respect to childbirth interventions? Are there any Strategic plans? Policies? |
| **Literature review** | 3. The literature review did not follow pyramid while discussing the previous findings (i.e. provide available evidence about the topic under study from global perspective to local context) |
| **Problem Statement** | 4. Missed the knowledge gap, what information is lacking/missing in the literature/what we can’t do because we don’t know the trends? (lack of the information to be provided by the present study) 5. What would we benefit from this study findings? (please state it clearly) |
| **Justification** | 6. Lack of information does not justify a study to be done. (please state clearly the benefits of the study findings; to whom, why and how?) |
| **Objectives** | 7. Specific objective 2 is not SMART, “*We also investigated how these variations are correlated both to each other, and to adverse neonatal and maternal outcomes, adjusted for maternal characteristics.”* (this is two in one, it can be separated to make it more specific) |
| **Methodology** | 1. What was the reason of focusing only on single births? 2. How was confounding controlled for? 3. Individuals from the same facility may have the same characteristics (may be clustered within the facility), how did you account for the clustering effect? |
| **Results** | 1. Childbirth interventions need to be clearly defined and clearly presented 2. Neonatal and maternal adverse outcomes should be clearly defined |
| **Discussion** | 1. Missed the comparisons between the study findings and available literatures (similarities & differences) 2. Missed the link of the results and possible explanations basing on what policies, interventions, changes in guidelines were in place at the time. |
| **Strengths and limitations** | 3. Did the missing values affect study results? if YES what could be the direction of bias? 4. Were there any forms of bias in the study? if YES how these might have influenced the present findings? 5. What is the contribution of the present study to the scientific community? |
| **Conclusion** | 6. The recommendations were not action oriented (i.e. state clearly, what recommendation, to whom with what action (taking into account the availability of resources): short term and long term action 7. What should be the way forward now that we have the study findings? |
