# Supplementary material for: Regional variations in childbirth interventions and their correlations with adverse outcomes, birthplace and care provider: A nationwide explorative study
Source: PLoS One. 2020 Mar 5;15(3):e0229488. doi: 10.1371/journal.pone.0229488 (PMC7058301; doi:10.1371/journal.pone.0229488)
Supplement: Attachment — Submitted filename: Response to reviewers.docx [file pone.0229488.s006.docx]

6 February 2020

Dear dr. Michael Johnson Mahande,

Please find enclosed our revised paper entitled ‘Regional variations in childbirth interventions and their correlations with adverse outcomes, birthplace and care provider: a nationwide explorative study’ (PONE-D-19-18857R1). We appreciate that we received the opportunity to revise our manuscript. We thank the reviewer for the constructive comments. We will address the suggestions one by one, referring to the line numbers. In the manuscript, changes are highlighted in red.

**Response to comments of Reviewer 2**

Introduction

- *I cannot find references number 22 and 23. Please add web address or other publication details.*

We added the web address of these two references.

Page 28, lines:

“22.	Perined. Perinatale Zorg in Nederland 2015 Utrecht: Perined; 2016 [Accessed 03-Feb-2020]. Available from: https://assets.perined.nl/docs/980021f9-6364-4dc1-9147-d976d6f4af8c.pdf.

23.	Stichting Perinatale Registratie Nederland. Perinatale Zorg in Nederland 2005 Utrecht: Stichting Perinatale Registratie Nederland; 2008 [Accessed 03-Feb-2020]. Available from: https://assets.perined.nl/docs/2f0a701a-d8f3-4cbd-98a1-2c4e588cf435.pdf.”

Methods

- *I would recommend using “RS” for the Spearman rank correlation coefficient. Use of “p-value” confuses readers, I think.*

We changed all “ρ-signs” into “rho” in the manuscript as well as in the table. We highlighted these changes in the manuscript.

Results

- *Again, confusing symbol used for the correlation coefficient.*

See our previous response.

- *Intrapartum referrals from midwife-led to obstetrician care seem very high, 55% to 68% for nulliparous women. Is this correct, are these referrals done during labour and birth? During active labour?*

These percentages are indeed correct. It concerns referrals between the start of labour and birth. The start of labour is defined as active uterine contractions or rupture of the membranes. The definition of intrapartum referral was described on page 8, lines 136-137 and the definition of onset of labour on lines 135-136:

“The onset of labour was defined as the onset of active uterine contractions or rupture of the membranes. Intrapartum referral to an obstetrician-led care setting was defined as a referral after the onset of labour and before birth.”

- *There is a misspelling in the title for figure 4 (aArtificial).*

We changed this accordingly.

Page 12, line 240:

“Fig 7. Interregional variation in Artificial rupture of membranes in midwife-led care at onset of labour”

- *In the tables, GR, DR, ZL etc need explanation, I suggest under the table.*

We added an explanation under the table.

Page 15, lines 247-248:

“GR=Groningen; FR=Friesland; DR=Drenthe; OV=Overijssel; FL=Flevoland; GD=Gelderland; UT=Utrecht; NH=Noord-Holland; ZH=Zuid-Holland; NB=Noord-Brabant; LB=Limburg. These regions are the twelve provinces in the Netherlands.”

Discussion

- *These results give an important message – wide variations in intervention rates depend to a large extent on different cultures in childbirth care – but I miss this message being highlighted in the conclusion. I would like to see the authors’ conclusions on referral rates.*

We added the following sentences into the conclusions:

Page 22, lines 416-419:

“High rates and large variations were found for intrapartum referral, indicating differences in risk perception and possibly in childbirth care culture between care providers. A correlation was found between intrapartum referral and PPH. More research is required into factors influencing care providers’ decision to refer a woman during labour.”

- *I am a little confused about the choice of subheadings in the Discussion section. First a summary, good, and the strengths and limitation, good. But what is meant by “Literature”? Is that a subheading or a sub-subheading after “Interpretation…”? I think this could be made clearer.*

We changed this sub-subheading into “Interpretation in view of previous literature” on page 18, line 321.

- *Subheading “Possible explanation….” seems to be about referral rates – maybe include “referral rates” in the subheading would make it easier to find those discussed. Thereafter the interventions studied have separate sub-headings so why not referrals?*

We changed this heading into “Possible explanations of variations in referrals and interventions” on page 20, line 360.

- *I understand that it is a little complicated to get all studied factors into the conclusion but I think it is worth a try.*

We added the following sentences into the conclusions:

Page 22, lines 416-419:

“High rates and large variations were found for intrapartum referral, indicating differences in risk perception and possibly in childbirth care culture between care providers. A correlation was found between intrapartum referral and PPH. More research is required into factors influencing care providers’ decision to refer a woman during labour.”

We are confident that our study is a first, important step in reducing the inappropriate use of medical interventions in maternal and newborn care, which is called for by important bodies, such as the WHO. We therefore hope that you will consider publication of our article.

We look forward to hearing from you.

Yours faithfully, on behalf of all authors,

Anna Seijmonsbergen-Schermers, MSc
